# Effect of Passive Stretching of Respiratory Muscles on Chest Expansion and 6-Minute Walk Distance in COPD Patients

**DOI:** 10.3390/ijerph17186480

**Published:** 2020-09-06

**Authors:** Asma Rehman, Jyoti Ganai, Rajeev Aggarwal, Ahmad H. Alghadir, Zaheen A. Iqbal

**Affiliations:** 1 Al Hosn One Day Surgery Center LLC, Al Sahel Tower Building, Post Box 37384, Abu Dhabi, UAE; asmaz84@gmail.com; 2 Department of Rehabilitation Sciences, Jamia Hamdard, New Delhi 110062, India; physio_jyoti@yahoo.co.in; 3 Neuro-Physiotherapy Unit, NSC, All India Institute of Medical Sciences, New Delhi 110029, India; physiorajeev@yahoo.co.in; 4 Rehabilitation Research Chair, College of Applied Medical Sciences, King Saud University, Riyadh 11433, Saudi Arabia; aha@ksu.edu.sa

**Keywords:** COPD, passive stretching, respiratory muscles, 6MWD, chest expansion, exercise rehabilitation

## Abstract

Background: Chronic obstructive pulmonary disease (COPD) is a major cause of morbidity and mortality worldwide. Hyperinflation of the lungs leads to a remodeling of the inspiratory muscles that causes postural deformities and more labored breathing. Postural changes include elevated, protracted, or abducted scapulae with medially rotated humerus, and kyphosis that leads to further tightening of respiratory muscles. As the severity of the disease progresses, use of the upper limbs for functional tasks becomes difficult due to muscle stiffness. There are various studies that suggest different rehabilitation programs for COPD patients; however, to the best of our knowledge none recommends passive stretching techniques. The aim of this study was to assess the effect of respiratory muscle passive stretching on chest expansion and 6-min walk distance (6MWD) in patients with moderate to severe COPD. Methods: Thirty patients were divided into two groups, experimental (*n* = 15) and control (*n* = 15). The experimental group received a hot pack followed by stretching of the respiratory muscles and relaxed passive movements of the shoulder joints. The control group received a hot pack followed by relaxed passive movements of the shoulder joints. Results: In the control group, there was no difference in chest expansion at the levels of both the axilla and the xiphisternum or in 6MWD between baseline and post treatment (*p* > 0.05). In the experimental group, chest expansion at the level of the axilla (*p* < 0.05) and 6MWD (*p* < 0.001) were significantly higher post treatment, while there was no difference in chest expansion at the level of the xiphisternum (*p* > 0.05). A comparison between control and experimental groups showed that chest expansion at the level of the axilla (*p* < 0.05) and 6MWD (*p* < 0.01) were significantly higher in the experimental group, while there was no difference in chest expansion at the level of the xiphisternum (*p* > 0.05). Conclusions: Although COPD is an irreversible disease, results of this study indicate that passive stretching of respiratory muscles can clinically improve the condition of such patients, especially in terms of chest expansion and 6MWD. Given the good effects of muscle stretching and the fact that such an exercise is harmless, clinicians and physiotherapists should consider including passive stretching of respiratory muscles in the rehabilitation plan of COPD patients.

## 1. Introduction

Chronic obstructive pulmonary disease (COPD) is a major cause of morbidity and mortality in the modern world [1]. According to the World Health Organization, 65 million people suffer from moderate to severe COPD, and at least 90% of COPD-related deaths occur in developing countries [2]. Estimates show that total deaths from COPD could increase by 30% in the next 10 years and it will become the third leading cause of death worldwide by 2030 [3]. Studies have reported the prevalence of COPD in India to be 3.49%, varying across different parts of the country [4,5].

Pathophysiology for people with COPD starts with damage to the airways and tiny air sacs in the lungs [6]. These airways become thick and inflamed, which destroys the tissues where oxygen is exchanged. The flow of air in and out of the lungs is decreased, which lowers the amount of oxygen reaching the body tissues. Further, getting rid of the waste gas carbon dioxide becomes difficult. This damage is irreversible [7].

Symptoms start with a cough with mucus and progress to dyspnea, which is a more frequent symptom of COPD [8]. The most typical finding in COPD patients is a persistent reduction in the ratio of forced expiratory volume in 1 s (FEV1) and forced vital capacity (FVC), which is requisite for the diagnosis of COPD [9]. Impaired exercise tolerance, muscle weakness, and poor quality of life are other common complaints in such patients, which can make them disabled and socially isolated [10]. Peripheral muscle weakness, deconditioning, and impaired gas exchange are important contributors to impaired exercise tolerance [11,12]. Hyperinflation of the lungs leads to remodeling of the inspiratory muscles, especially the diaphragm, which may become depressed and its movement is reduced [13,14]. It also leads to postural deformities and increased labor in breathing [15]. Postural changes include elevated, protracted, or abducted scapulae, with medially rotated humerus, and kyphosis, which leads to further tightening of the respiratory muscles [16,17]. As the severity of the disease progresses, use of the upper limbs for functional tasks becomes difficult due to muscle stiffness [18,19].

There are various studies that suggest different rehabilitation programs to improve aerobic capacity and chest wall mobility for COPD patients [18]. According to the American College of Chest Physicians, standard pulmonary rehabilitation programs consisting of aerobic physical conditioning exercises, despite other benefits, have not been shown to modify lung function [19,20]. Some of these programs include active stretching techniques to be done by patients themselves; however, there are fewer studies that have used passive stretching techniques for such patients [21,22,23,24]. Moreover, the American Thoracic Society guidelines for pulmonary rehabilitation do not recommend stretching of respiratory muscles in such cases [18,25,26]. This study was conducted to investigate whether passive stretching of respiratory muscles can benefit COPD patients. We tested the hypothesis that passive respiratory muscle stretching can improve chest expansion and 6-min walk distance in COPD patients.

## 2. Materials and Methods

### 2.1. Patients

This study was done in a chest specialty hospital. Thirty five stable COPD patients were invited to participate in this study. Inclusion criteria were age between 40–45 years, and moderate to severe COPD as defined by the Global Initiative for Chronic Obstructive Lung Disease (GOLD) guidelines, i.e., FEV1/FVC < 0.7 and FEV1 % pred. 30–80 [27]. Subjects were excluded if they had any other associated respiratory problems, cardiac disease, or neuromuscular disease. Thirty patients who passed the study criteria were asked to sign an informed consent form before participating in the study. Ethical approval was obtained from the Institutional Review Board (IRB) according to the Declaration of Helsinki.

### 2.2. Procedure

Subjects were divided into 2 groups, experimental (*n* = 15) and control (*n* = 15). The experimental group received a hot pack followed by stretching of respiratory muscles and relaxed passive movements of the shoulder joints. The control group received a hot pack followed by relaxed passive movements of the shoulder joints.

### 2.3. Intervention

Hot pack: An electrical hot pack sized 35.5 × 68.5 cm was used bilaterally to heat the shoulders of the subjects in both experimental and control groups. The temperature was set at 63 °C. The subjects were informed that they should feel a comfortable level of warmth throughout the session. The temperature was adjusted if they felt excessive heat. Each session lasted for around 10 min [28].

Muscle stretching: Passive stretching exercises consisted of sternocleidomastoid, scalene (anterior, medius, and posterior), trapezius (descending part), pectoralis major (clavicle, sternal, and abdominal part), pectoralis minor, internal and external intercostal muscles, and diaphragm muscles as described below. All muscle stretching exercises were performed bilaterally by a trained physical therapist. Subjects were placed in a supine or side lying position. Their knees were bent to correct the lumbar lordosis, and any postural compensations were avoided. Stretching was done in the expiratory phase, except for the intercostal muscles. There were two sets of ten repetitions for each muscle with a one-min interval between them [29,30]. The complete treatment session lasted for approximately 60 min for 5 days [23,30,31,32].

Sternocleidomastoid: Subjects were made to lie in a supine position with side flexion and rotation of the head to the opposite of the side being stretched. The therapist placed one of his hands on the occipital region and the other on the sternal region. The sternal region was displaced in the cranial–caudal direction [29].

Scalene: Subjects were made to lie in a supine position. The therapist placed one hand on the occipital region with the other on the sternum region, to promote displacement of the two points in opposite directions [29].

Trapezius (descending part): Subjects were made to lie in a supine position with side flexion of the head to the opposite of the side being stretched. The therapist supported the occipital region with one hand and the shoulder with the other hand, such that it caused displacement of two points in the cranial–caudal direction [29].

Pectoralis major: Subjects were made to lie in a supine position, with the arm abducted on the side to be stretched, such that the forearm was flexed and the hand resting on the occipital region. The therapist performed displacement with one hand on the upper third of the arm and the other hand on the lateral side of the upper chest in the direction of muscle fibers [29].

Pectoralis minor: Subjects were made to lie in a supine position with arms by their sides. The therapist stabilized the scapula and humeral head with one hand, while the other hand palpated medially into the proximal axilla, proceeding superiorly towards the coracoid process to allow his fingers to be fixed posterior to the proximal end of the pectoralis minor muscle. He then applied pressure in the anterior direction, thus applying tensile force directly to the muscle [33].

Intercostals: Subjects were made to lie in a lateral decubitus position over a half moon-shaped foam roller placed in the infra-axillary region, with their forearms flexed and hands resting on the occipital region. The therapist used the palmar region of both hands to mobilize the ribs in the cranial–caudal direction [34]. A side stretch was performed in this position at the moment of inspiration, and the ribs were monitored during expiration [35].

Diaphragm: Subjects were made to sit erect. The therapist stood behind the subject and passed his hands around the thoracic cage, introducing his fingers into the subcostal margins. The subject’s trunk was slightly rounded in order to relax the rectus abdominis muscle. As the subject exhaled, the therapist eased his hands caudally to grasp the lower ribs at the subcostal margin. Firm but gentle traction was maintained as the patient inhaled [36,37].

Relaxed passive movements of the shoulder joint complex: flexion–extension (sagittal plane), abduction–adduction (coronal plane), medial–lateral rotation (transversal plane), horizontal abduction–adduction (transversal plane), scapular protraction–retraction, (movement toward the spine), elevation–depression (movement upward and downward), and upward–downward rotation [38].

### 2.4. Outcome Measures

Chest expansion and 6-min walk distance (6MWD) were measured before the start of the treatment and after completion of 5 sessions in both the groups. Chest expansion was measured in centimeters at the level of the axilla and the xiphisternum using a tape. Each measurement was obtained after maximal expiration followed by maximum inspiration [39,40]. The 6MWD was calculated according to American Thoracic Society guidelines. It was performed indoors on a long, flat, and straight hard surface. The walking course was 30 m in length, and it was marked every 3 m. The points where the patient had to turn around were marked with a bright color. Subjects were instructed to walk and cover as much distance as possible within 6 min. The distance covered was measured in m [41,42]. The clinically relevant minimum difference in 6MWD as presented in previous studies is 25–35 m [43].

### 2.5. Statistical Analysis

Data analysis was done using SPSS software for windows version 18 (Statistical package for the social sciences, IBM Inc., Amonk, NY, USA). A pre-/post-test experimental design was used for this study. Percentage predicted analysis was used for both outcome measures. A paired t-test was used to analyze the difference between pre and post intervention outcome measures within the groups. An unpaired *t*-test was used to find the difference between the groups. The results were considered significant if the *p*-value was less than 0.05.

## 3. Results

Demographic characteristics of the subjects are given in Table 1. There were no significant differences (*p* > 0.05) in age, height, weight, BMI, or predicted FEV1 between control and experimental groups at baseline (Table 1). There were no differences in baseline values of chest expansion and 6MWD between the two groups (*p* > 0.05). 

### 3.1. Comparison within the Groups

Control group: Chest expansion at the level of the axilla decreased by 0.05 cm after intervention. On the other hand, chest expansion at the level of the xiphisternum and 6MWD had increased by 0.09 cm and 9.45 m, respectively, after intervention. There was no difference in chest expansion at the levels of both the axilla and the xiphisternum or in 6MWD between baseline and post treatment (*p* > 0.05) in the control group (Table 2).

Experimental group: Chest expansion at the level of the axilla and the xiphisternum, as well as 6MWD had increased after intervention by 0.46 cm, 0.23 cm, and 35.04 m, respectively. However, chest expansion at the level of the axilla was significantly higher post treatment (*p* < 0.05), while there was no difference in chest expansion at the level of the xiphisternum between baseline and post treatment (*p* > 0.05). The 6MWD was also significantly higher (*p* < 0.001) post treatment (Table 3).

### 3.2. Comparison between the Control and Experimental Groups

Chest expansion at the level of the axilla was significantly higher in the experimental group (*p* < 0.05), while there was no difference in chest expansion at the level of the xiphisternum between experimental and control groups (*p* > 0.05). The 6MWD was also significantly higher (*p* < 0.01) in the experimental group (Table 4).

## 4. Discussion

The aim of this study was to study the effect of respiratory muscle passive stretching on chest expansion and 6-min walk distance in patients with moderate to severe COPD. Results show that there was significant improvement in chest expansion at the level of the axilla and 6MWD after the treatment in the experimental group. This difference was also significant in comparison to that of the control group. To the best of our knowledge, this is one of the few studies that have used passive stretching of respiratory muscles in COPD patients.

Improvement in chest expansion was greater at the level of the axilla than at the level of the xiphisternum. Stretching of inspiratory muscles does not affect lung structure; hence, this increase in chest expansion could be due to improved chest wall mobility. Increased mobility of the chest wall is often nonsynchronous with abdominal motion either due to weakness of the diaphragm or its increased excursion [44,45]. A previous study reported immediate improvement of the respiratory pattern following respiratory muscle stretch gymnastics in COPD patients, which was attributed to afferent information from respiratory muscles [23]. Another study reported increased chest expansion following chest wall stretching exercises [25].

There was a significant increase in 6MWD for the experimental group post respiratory muscle passive stretching, as well as in comparison to the results of the control group. Previous studies report that such increases in the distance covered by different pulmonary disease patients is due to changes in ventilator capacity and chest wall compliance, improved respiratory pattern, and decreased chest wall stiffness [22,46,47,48]. This can also be due to increased chest expansion [22], which was also seen in our study. Different exercises can alleviate exercise-induced imbalance in pulmonary ventilation, lowering the respiratory threshold and indirectly increasing 6MWD [21]. The 6MWD is frequently used in research to measure functional exercise capacity [48].

Alteration of head and neck positions, such as in individuals with forward head posture and torticollis, has been shown to have a negative impact on respiratory function, which should be considered during assessment in order to reduce the tension on the respiratory system and avoid associated consequences [49]. Stretching of respiratory muscles has been shown to improve FVC [19,50]. Another study reported that stretching of respiratory muscles during passive chest recoiling improves their length, mobility, and power, as well as lung capacity, which further improves ventilation and oxygenation [25]. Stretching techniques are simple to execute and do not require any costly equipment. Moreover, they are harmless and the patient can adapt to them easily as they can be performed in a restricted area, hence they have potential to be included in pulmonary rehabilitation programs.

### Limitations

This study was performed on a small sample, and spirometry data alone cannot describe the full picture of COPD patients. We propose a similar large-scale study considering data on symptoms, medication, exacerbation, smoking, gender, and other covariates to further confirm the findings of the study.

## 5. Conclusions

Although COPD is an irreversible disease, the results of this study indicate that passive stretching of respiratory muscles can clinically improve the condition of such patients, especially in terms of chest expansion and 6MWD. Given the good effects of muscle stretching and the fact that such an exercise is harmless, clinicians and physiotherapists should consider including passive stretching of respiratory muscles in the rehabilitation plans of COPD patients.

## Figures and Tables

**Table 1:** Demographic data: experimental and control groups, *n* = 15 each (mean ± SD).

Variable	Control Group	Experimental Group	*p*-Value
Age (years)	53.53 ± 8.12	53.13 ± 8.47	0.08
Height (cm)	161.73 ± 5.98	161.73 ± 5.40	1.00
Weight (kg)	52.13 ± 7.03	59.06 ± 11.41	0.05
BMI (kg/m^2^)	20.11 ± 6.50	22.78 ± 8.40	0.06
FEV1 (%) predicted	56.53 ± 14.82	49.60 ± 12.68	0.18

**Table 2:** Comparison of pre and post intervention chest expansion and 6MWD in the control group: Mean ± SD.

Variable	Pre Intervention	Post Intervention	*p*-Value
Chest expansion: Axilla (cm)	2.75 ± 1.08	2.70 ± 0.92	0.61
Chest expansion: Xiphisternum (cm)	2.42 ± 0.89	2.51 ± 0.70	0.49
6MWD (m)	495.95 ± 80.02	505.40 ± 75.04	0.65

**Table 3:** Comparison of pre and post intervention chest expansion and 6MWD in the experimental group: Mean ± SD.

Variable	Pre Intervention	Post Intervention	*p*-Value
Chest expansion: Axilla (cm)	2.15 ± 0.78	2.61 ± 0.80	0.003 *
Chest expansion: Xiphisternum (cm)	1.77 ± 0.69	2.00 ± 0.77	0.012
6MWD (m)	498.43 ± 82.52	533.47 ± 78.37	0.0001 *

* significant, *p* < 0.05.

**Table 4:** Comparison of mean difference in pre and post intervention chest expansion and 6MWD between the control and experimental groups: mean ± SD.

Variable	Control Group	Experimental Group	*p*-Value
Chest expansion: Axilla (cm)	−0.05 ± 0.16	0.46 ± 0.02	0.017 *
Chest expansion: Xiphisternum (cm)	0.09 ± 0.19	0.23 ± 0.08	0.42
6MWD (M)	9.45 ± 4.98	35.04 ± 4.15	0.0001 *

* significant, *p* < 0.05.

## Data Availability

The datasets used in this study are available from the corresponding author on request.

## Abbreviations

COPD	Chronic obstructive pulmonary disease
FEV1	Forced expiratory volume in 1 s
FVC	Forced vital capacity
GOLD	Global Initiative for Chronic Obstructive Lung Disease
6MWD	6-min walk distance
